# Evaluation of hemodialysis vascular access involving multidisciplinary integration: Perspective from Latin America and Peru

**DOI:** 10.3389/fneph.2022.1051541

**Published:** 2023-01-05

**Authors:** Edwin Castillo Velarde, José A. Ruiz-Peñafiel, Sheyla Alfaro Ita, Tushar J. Vachharajani

**Affiliations:** ^1^ INICIB, Instituto de Investigación de Ciencias Biomédicas, Universidad Ricardo Palma, Lima, Peru; ^2^ Peruvian Association of Vascular Access (APDAV), Lima, Peru; ^3^ Department of Nephrology, Hospital Guillermo Almenara, Lima, Peru; ^4^ Department of Cardio-Vascular Surgery, Hospital Guillermo Almenara, Lima, Peru; ^5^ Department of Radiology, Hospital Guillermo Almenara, Lima, Peru; ^6^ Cleveland Clinic Lerner College of Medicine of Case Western Reserve University, Cleveland, OH, United States; ^7^ Department of Kidney Medicine, Glickman Urological & Kidney Institute, Cleveland, OH, United States

**Keywords:** vascular access, hemodialysis, multidisciplinary approach, Latin-America, Perú

## Abstract

The perspective of vascular access care in patients with end-stage renal disease has migrated from nephrology-centered or vascular surgery-centered care to multidisciplinary-focused patient-centered care. This new perspective should not only be theoretical but also have practical utility. A non-multidisciplinary focus can contribute to the low prevalence of arteriovenous fistula (AVF) in the population. Latin America has multiple health systems and the coordination of vascular access is heterogeneous. In Peru, there is a high prevalence of central venous catheter use with its associated complications, such as stenosis, thrombosis, infection, and recurrent hospitalizations in the context of fragmented care. However, in the last few years, there has been an effort to integrate the communication between vascular surgery, interventional radiology, and nephrology to improve vascular access care. In this review, we analyze the availability of care, the intervention, and the future directions from the experience of both perspectives.

## Introduction

Dialysis vascular access care involves several steps. A constant and reliable nephrology service in a hospital and/or outpatient setting is required, and the continuity of dialysis of the patient depends on available vascular access. It also involves an early nephrology referral for evaluation/creation of arteriovenous fistula during stage 4 chronic kidney disease. Where a patient has a dysfunction of the arteriovenous fistula during dialysis, their outpatient dialysis center refers the patient to the hospital. These patients are treated with thrombectomies by acute thrombosis or angioplasties, by juxta-anastomotic stenosis, or require placement of a central venous catheter. When the patient is admitted, treatment and planning of convenient dialysis is performed by the nephrology service. The interventional radiology and vascular surgery groups intervene for rescue or corrective procedures in vascular access. This reality is repeated in many contexts around the world. The future coordination and planning of vascular access for patients are undertaken by the nephrologist with a multidisciplinary approach (1), and the recommendations for patients should be individualized and include patient-centered shared decision-making.

## Health system and reports of vascular access in Latin America and Peru

Peru has a population of 32.5 million (2019), with a prevalence of dialysis-dependent chronic kidney disease of 974 per million inhabitants (pmp). The renal transplant rate is 4 pmp, which is significantly lower than other countries in Latin America (e.g., Brazil, 28 pmp; Uruguay, 43 pmp) (2–4). In Peru, there are 1.7 nephrologists per million inhabitants, and in Latin-America it has been reported to be 19 per million in 2019 [the recommendation of the Pan-American Health Organization (PAHO) is 20 pmp] (5, 6). In 2022, the Peruvian Medical College reported 565 nephrologists, 302 thoracic and cardiovascular surgeons, and 13 interventional radiologists pmp (7). Therefore, there is a small number of specialists involved in vascular access in Peru.

In Peru, hemodialysis (85.9%) is the predominant renal replacement modality, with the central venous catheter (CVC) the most frequently used method for vascular access (2, 8). Compared with Latin America, the use of the native arteriovenous fistula (nAVF) in the Peruvian population remains low (24%), as shown in **Table 1**.

**Table 1:** Epidemiology of vascular access in prevalent dialysis patients in Latin-America.

Country	nAVF	pAVF	Tunneled catheter	Non-tunneled catheter
Argentina^a^	67.9%	12.3%	8.7%	11.1%
Uruguay^b^	47.6%	23.2%	20.7%	8.4%
Peru^c^	24%	–^*^	53%	23%

nAVF, native arteriovenous fistula; pAVF, prosthetic arteriovenous fistula.

a. Registro Argentino de Diálisis Crónica 2020. Instituto Nacional Central Único Coordinador de Ablación e Implante (INCUCAI) y Sociedad Argentina de Nefrología (SAN) (https://www.cadradialisis.org.ar/registro.php).

b. Registro Uruguayo de Diálisis 2018. Sociedad Uruguaya de Nefrología (http://nefrouruguay.org.uy/registro-de-dialisis/).

c. Registro Nacional de Diálisis (Rendes) 2016. Hospital Guillermo Almenara.

*Not reported.

Peru has a tripartite health system: public (61%), social security (33%), and private (4%). In addition, as in other Latin American countries (e.g., Argentina, Bolivia, Ecuador, Salvador, Guatemala, Haiti, Honduras, México, Nicaragua, Panama, Paraguay, and Venezuela), Peru does not have universal health coverage, but is fragmented. Few countries have an integrative system, such as those in Brazil, Chile, Colombia, Costa Rica, Cuba, Dominican Republic (in process), and Uruguay (9, 10). According to a report from the WHO (World Health Organization), Peru ranks 129th in the world in the overall level of population health, and Colombia, Chile, Costa Rica, and Cuba are rated the highest among the Latin American nations (11). In Peru, the healthcare cost per capita was $626 in 2016, including the cost of the main health systems, of which $199 is provided by social security, and $132 by the public system (the remaining cost is private and equates to $103 out of pocket). In Chile, the health per capita cost is $2182; in Uruguay it is $2102 and in Costa Rica it is $1285. Therefore, the health cost in Peru is lower, with a diverse health system that contributes to heterogeneous care in vascular access (12). A strategized plan is needed to homogenize and optimize the distribution of medical care for achieving the objectives in vascular access.

In 2016, the Peruvian Social Security (EsSalud) reported 10,710 patients being treated with dialysis, with 7,778 receiving coverage from public health insurance (SIS, “seguro integral de salud”) (2, 8). The problem of vascular access for dialysis patients involves higher costs because of complications related to central venous catheter blood stream infection and central venous occlusion requiring endovascular intervention. These complications are the most frequent causes of admission in the nephrology service at Hospital Guillermo Almenara in Lima, Peru, a national referral hospital for social security. This reality requires corrective actions that consider the segmented health system and low health cost in Peru and other Latin American countries.

## Perspective: Non-multidisciplinary

In a fragmented view related to the patient, a nephrologist does not consider the preferences of the patient and the decision is unilateral. The medical advice in vascular access promotes the arteriovenous fistula more than the central venous catheter, in order to avoid complications such as sepsis (fivefold to tenfold increased risk) and death (twofold to threefold increased risk) (13, 14). However, if the preferences of the patient are not taken into account during decision-making, it is not possible to properly plan vascular access. The patient should participate, for example, during the predialysis and preoperative periods to preserve their veins, understand concepts such as flow of fistula, and ask about the need for assessment by Duplex ultrasound (15, 16). Hence, empowerment of the patient is required to allow the planning and monitoring of vascular access.

In a fragmented view related to other specialties, the nephrologist is responsible only for the processes that take place inside their service. For example, on vascular access, checking only the placement of the central venous catheter and not creating an arteriovenous fistula, given that it depends on the availability of the vascular surgery department. Many countries share this problem and also have a low prevalence of arteriovenous fistula, with no more than 67% within 3 months of dialysis. Moreover, in the incident population, more than 80% did not have a nAVF. This is a traditional perspective with high cost because the typical causes of hospitalization in a nephrology service include catheter-related bloodstream infection and central venous occlusion. This view considers the distance between vascular access indicators such as time catheter dependence dialysis and does not consider the effects of use a catheter before nAVF creation, such as in central venous occlusion, or the effects of reducing the patency of nAVF in the future. Without changes that promote the relationship between nephrology and vascular surgery, this perspective promotes only the same indicators, consequences, and costs, and infringes the ethical principle of beneficence for the patients.

Some circumstances may maintain this resigned perspective. First, a referral and counter-referral patient management system. Late referrals to nephrology or a vascular surgeon are typical reasons to delay the creation of nAVF. Second, vascular surgery that considers nAVF as a surgical procedure of low priority. Regarding nephrology, the creation of nAVF is a surgery priority because in some countries, such as Peru, the prevalence of nAVF is low (24%). Besides, the performance of this procedure is centralized in cities such as Lima (Peru), where the prevalence of hemodialysis in the population is more than 50% and the demand for care of patients with end-stage chronic renal disease converges in only three hospitals in the security social health system. For reference, the incident hemodialysis population in one national referral hospital is 40 patients per month and, according to international guidelines, AVF creation should be at least 67%. Therefore, in this case, 27 patients per month would require attention by vascular surgeons for two to three shifts per week. This requirement contrasts with the priority of vascular surgery in the same hospital for procedures such as coronary revascularization, femoral-popliteal bypass, and valve replacement surgery.

A non-multidisciplinary focus contributes to the low prevalence of arteriovenous fistula (AVF) in the population and consequently affords high cost related to the use of CVC. A preliminary transversal report on the costs of nAVF and CVC in Hospital Guillermo Almenara, Lima, Peru, in 2021 (20 patients in each group during 5 months of follow-up) evidences three times more costs related to CVC than AVF ($41,684 vs. $14,418), as depicted in **Table 2**.

**Table 2:** Comparative costs between catheter venous central (CVC) and arterio-venous fistula (AVF).

	CVC group (*n* = 20)	AVF group (*n* = 20)
**Age (years)**	62.4	57.3
**Sex (female)**	50%	50%
**D**i**al**y**sis duration (months)**	5.25	9.45
**CVC use duration (months)**	5.35	5.85*
**Functional nAVF use (months)**	N/A	3.64
**Hospitalizations related to vascular access (events)**	5	1
**Deaths related to vascular access (events)**	3	0
**Hospitalizations non-related to vascular access (events)**	10	8
**Deaths non-related to vascular access (events)**	5	1
Cost		
**Placement/re-placement CVC ($/.)**	7,580.58	5,540.94*
Other service related to vascular access** ($/.)	976.756	242.65
**Creation nAVF ($/.)**		4,848.26
**Hospitalizations related to vascular access ($/.)**	33,131.40	3,788.88
**Total cost ($/.)**	41,689.04	14,418.40

*CVC previous to nAVF creation. **Includes thrombolysis therapy. Data from Hospital Guillermo Almenara, Lima, Peru. NA, Not applicable.

In a fragmented view related to interventional radiology, the patient is referred from nephrology, for placement of a central venous catheter under fluoroscopy guidance, without any future plan of vascular access. For example, if the patient has a diagnosis of only dysfunction of the central venous catheter, interventional radiology allows placement of the catheter in a permeable location, which is better if the procedure requires less time. In this perspective, the end point is placement of a catheter. However, a different perspective considers the planned AVF. The AVF should be planned by echography and clinical criteria and, consequently, the interventional radiologist would avoid ipsilateral placement of a catheter in planned AVF. This process sometimes requires endovascular procedures to permeabilize central venous stenosis before AVF creation.

When patients start hemodialysis, many of them have a central venous catheter before AFV. Some of them will have central venous occlusion, so require a cacography before nAVF creation. However, many nephrology departments do not have c-arm fluoroscopy, or the required training to use it, so the demand for interventional radiology increases. For the reality of a national referral hospital in Peru, some procedures related to interventional radiology are highly demanding, such as percutaneous drainage, biopsy, and embolization for hemorrhage; therefore, deferral to nephrology occurs for all vascular access procedures such as tunneled catheter placement, angioplasty, or thrombectomy.

To sum up, in the case of interventional radiology and vascular surgery, demand exceeds supply. A non-multidisciplinary perspective only contributes to the gap between problem and solution.

## Perspective: Multidisciplinary patient-centered

If we are aware of limitations in the health system, the number of specialists, and the demand for vascular access, we need to change the perspective toward integrative coordination between nephrology, vascular surgery, and interventional radiology. In many countries, the priority for nephrology is the creation of nAVF because there is a low prevalence of AFV and a high prevalence of complications related to CVC. Some processes of the health system are difficult to change, such as referral time; therefore, we first need to start planning of vascular access from the moment the patient begins their care in nephrology. The care of vascular access for the patient frequently starts in hemodialysis with a central venous catheter; the time from this point until the creation of nAVF is very important because a long time is dissuasive for accepting the creation of nAVF, despite educational efforts. The first step starts with an interview between the nephrologist and the patient in the outpatient clinic or hemodialysis unit. In this step, clinical evaluation is important (e.g. Allen Test) but fails to differentiate the subclinical pathology; thus, ultrasound evaluation of vascular access is essential. The ultrasound should be performed by a nephrologist dedicated to vascular access, and, according to demand, ultrasound availability is required every day. It is not possible to plan proper vascular access in nephrology without parameters such as cephalic and arterial diameter, permeability, calcifications, velocity, and sites of potential anastomosis. At this point, integration with vascular surgery is important because most patients require radiocephalic nAVF as an outpatient procedure, but some patients require special coordination to plan a basilic vein transposition or brachiocephalic fistula in co-morbid inpatients. Nephrologist and nurse coordinators are needed to manage this multidisciplinary process through successful communication.

An integrative view, together with vascular surgery, allows the continuous monitoring of AVF to promote patency. The ultrasound is performed at 6–8 weeks from AVF creation, with prior cannulation to verify the maduration. Frequent monitoring allows early identification of stenosis or deficient flow. These findings prevent late actions and potential loss of AVF, and promote patency with procedures such as re-anastomosis by vascular surgery. In a previous study (17), the monitoring of vascular access blood flow reduced the morbidity and related costs due to decreased numbers of hospitalizations, catheters placed, and surgical interventions.

An integrative view with interventional radiology promotes communication on equipment usage and improves the vascular access plan for the patient. Elective procedures in the cases of catheter dysfunction or thoracic central vein occlusion syndrome are planned considering the priority of AVF creation, and patency is also promoted if AVF was already created. If the case is complex, for example, exhausted vascular access or thoracic central vein occlusion type 4, a multidisciplinary discussion is scheduled to analyze the endovascular strategies.

It has been reported that a multidisciplinary approach on vascular access increased nAVF creation from 33% to 69%, reduced surgical complications, and promoted the patency, making the framework more cost-effective (18). Multidisciplinary equipment shares the same goal, planning the best option for vascular access. Although in our context the promotion of radiocephalic nAVF is a priority, management is individualized. In some patients, the best option is placement of a tunneled central venous catheter; other patients require closing of the AVF when there is a high flow and pulmonary hypertension.

## Peruvian experience in integrative perspective

It was not easy to change the perspective of physicians involved in vascular access care such as vascular surgery or interventional radiology, starting from the same nephrology. Thus far, changes have been progressive. The first condition for this integrative experience is to determine the vascular access equipment workflow. Not all vascular surgeons, interventional radiologists, and nephrologists are interested in vascular access. In countries such as Japan where the AVF prevalence is over 90%, physicians from other specialties participate in the creation of AVF. Urologists and surgeons involved in dialysis are integrated into the workflow of the vascular access group (19). The goal is to supply the demand with an integrative view. In the case of interventional nephrology, endovascular management meets the needs of the patients; hence, there is an opportunity for training in this subspecialty. However, in a country with a disproportion of specialties, as we have mentioned above, the demand exceeds supply. Consequently, work and effort need to be distributed appropriately. From interventional nephrology and interventional radiology, it is possible to make many differentiating procedures. An interventional nephrologist can perform procedures such as cacography or phlebography whenever necessary, or fistulography for detecting AVF stenosis. It is even possible to treat AVF stenosis with angioplasty guided by echography, which is convenient for many nephrology departments if they do not have fluoroscopy. Thus, it is possible to improve the planning of vascular access, promoting the patency of AVF. Integrative work with interventional radiology is required for some complex clinical cases of central venous occlusion.

The second condition for the integrative experience in the vascular access group is communication. Many clinical cases can be discussed to explore alternative options for vascular access under the perspectives of vascular surgery, interventional radiology, and nephrology.

In the Peruvian experience, at Hospital Guillermo Almenara, some nephrologists involved in vascular access group have been trained in ultrasonography; the first step to planning an AVF is clinical and echographic evaluation. This strategy allows the demand to be supplied, allows selection, promotion, and creation of distal nAVF, and reduces the referral delay for vascular surgery. This outpatient procedure is elective. On the other hand, nephrologists select patients with special surgery conditions. Impatient hospitalization is performed in the nephrology department, with a previous evaluation provided from a vascular surgeon involved in the vascular access. In 2021 409 ultrasounds were performed by vascular surgeons, with 110 arteriovenous fistulas produced. In the first six months of 2022, 459 ultrasounds were performed by nephrologists.

## Conclusion

An integrative multidisciplinary perspective in vascular access is fundamental to optimize the supply of a continuous demand in patients requiring creation of arteriovenous fistula, or to promote their patency. Appropriate distribution of responsibilities can reduce the drawbacks of segmented health systems or reduced numbers of specialists. Determining vascular access equipment workflows and promoting continuous communication are essential conditions for this new perspective in nephrology.

## Data availability statement

The original contributions presented in the study are included in the article/supplementary material. Further inquiries can be directed to the corresponding author.

## Author contributions

EC: Manuscript draft, review of literature, and final version; JR-P: manuscript draft; SA: manuscript draft; TV: Mentor and final version. All authors contributed to the article and approved the submitted version.
